# Supplementary material for: Agreement between Family Members and the Physician’s View in the ICU Environment: Personal Experience as a Factor Influencing Attitudes towards Corresponding Hypothetical Situations
Source: Healthcare (Basel). 2023 Jan 25;11(3):345. doi: 10.3390/healthcare11030345 (PMC9914929; doi:10.3390/healthcare11030345)
Supplement: Supplementary file 1 [file healthcare-11-00345-s001.zip › healthcare-2177996-supplementary.pdf]

Supplementary Material

Supplementary Table S1. Proximity matrix of the answers in the four questions of the two hypothetical scenarios (Dice measure)[1]

		Scenario 1 Question 1				Scenario 1 Question 2				Scenario 2 Question 1			Scenario 2 Question 2		
		Intensivists	Family	Patient	Uncertain	Intensivists	Family	Patient	Uncertain	Intensivists	Family	Uncertain	Shouldn't stop	Intensivists	Family
Scenario 1 Question 1	Family	.000													
	Patient	.000	**.600**												
	Uncertain	.000	.000	.000											
Scenario 1 Question 2	Intensivists	.220	**.632**	.535	.059										
	Family	.062	**.749**	.585	.020	**.746**									
	Patient	.062	.569	**.877**	.061	.515	.583								
	Uncertain	.000	.094	.062	**.706**	.000	.000	.000							
Scenario 2 Question 1	Intensivists	.581	.000	.078	.182	.194	.060	.135	.171						
	Family	.068	**.733**	**.784**	.067	**.630**	**.667**	**.763**	.106	.000					
	Uncertain	.000	.130	.167	.444	.132	.155	.176	.368	.000	.000				
Scenario 2 Question 2	Shouldn't stop	.148	.158	.112	.069	.162	.167	.140	.065	.133	.137	.061			
	Intensivists	.177	.578	.597	.139	**.670**	.560	.595	.154	.190	**.681**	.151	.000		
	Family	.106	**.670**	**.730**	.104	.598	**.627**	**.709**	.118	.074	**.829**	.130	.000	**.774**	
	Uncertain	.053	.107	.206	.350	.127	.131	.214	.333	.146	.166	.364	.000	.000	.000

(1) Similarities larger than 0.6 are bold. Lower than 0.3 are grayed out.
